# Bridging complexity through integrative systems neuroscience

**DOI:** 10.3389/fsysb.2024.1487298

**Published:** 2024-11-06

**Authors:** Eric H. Chang

**Affiliations:** ^1^ Institute for Bioelectronic Medicine, Feinstein Institutes for Medical Research, Northwell Health, Manhasset, NY, United States; ^2^ Donald and Barbara Zucker School of Medicine at Hofstra/Northwell, Hempstead, NY, United States; ^3^ Elmezzi Graduate School of Molecular Medicine, Manhasset, NY, United States

**Keywords:** systems neuroscience, connectome, multidisciplinary approaches, multiscale, emergent properties, computational modeling, omic analysis

## Introduction

Neuroscientists have traditionally taken a reductionist approach to understanding the immense complexity of nervous systems. As is the case in other fields of biology, the method of reducing nervous systems into their constitutive parts has proven useful for understanding neural circuits and how they function. As a result, modern neuroscience has thrived on cataloging and scrutinizing individual components of complicated neural systems. However, substantial gaps persist in understanding how these disparate components connect and interact to generate higher-order functions. Bridging these gaps requires a concerted effort to integrate knowledge across sub-fields in neuroscience and, more broadly, across biology. Systems biology is a scientific approach used to examine complex biological processes at the level of systems, rather than focusing on individual discrete parts (Kitano, 2002; Mesarovic, 1968). A “system” is a group of mutually dependent components that work together to form a unified whole. The goal of a systems approach is to understand a holistic big picture in the context of integrated systems that are dynamic and interrelated. By taking a systems biology approach to understanding the nervous system, we can attempt to integrate and understand interactions between the different neural components that give rise to higher-order emergent phenomena (Geschwind and Konopka, 2009; Grillner et al., 2005).

The struggle between understanding individual parts and the larger whole has been a part of neuroscience since its origin as a scientific discipline. Over a century ago, the field was shaped by the opposing theories of two leading neuroanatomists, Santiago Ramón y Cajal and Camillo Golgi. On the one hand, Golgi’s *reticular doctrine* posited that the nervous system was an interconnected nerve network (“a large syncytium”) that was seamless and continuous (Glickstein, 2012). In contrast, Cajal proposed the *neuron doctrine* which stated that individual nerve cells were the basic structural and functional units of the nervous system (Cajal, 1888; Cajal, 1899). The structural evidence from the microscopes and stains available to scientists at the time supported Cajal’s neuron doctrine. In fact, it was actually Golgi’s *la reazione nera* or “black reaction” (now known as the Golgi stain) that produced the most convincing structural evidence that neurons were structurally separated elements. The introduction of the electron microscope in the 1940s definitively demonstrated that neurons were not continuous but were instead distinct entities separated by synapses with extracellular space in between them (Palay, 1956; Porter et al., 1945). While both Ramón y Cajal and Golgi were awarded the Nobel Prize in 1906 for their work on the structure of the nervous system, it was Ramón y Cajal who would widely be considered as the founder of modern neuroscience, and his neuron doctrine has long served as a foundation for the field.

Perhaps because of this foundation on the neuron doctrine, many of the workhorse techniques and methods in modern neuroscience have been catered to investigating individual components that make up neural circuits. For example, Golgi stains and patch-clamp electrophysiology highlight individual neurons. This conceptual focus on individual neurons has led to a compartmentalization of knowledge that has obscured, to some extent, our ability to integrate data on how individual functions enable higher-order processes (Yuste, 2015). Moreover, the reductionist bias and a reliance on big data or methods-driven approaches in neuroscience has left us with many descriptions, but few explanations (Krakauer et al., 2017). As a result, what is generally lacking in the field are accepted theories of nervous system function that explain how individual neurons or groups of neurons (e.g., circuits) contribute to neural systems that then give rise to behavior, cognition, or other emergent properties of nervous systems.

### Integrative systems neuroscience: mind the gaps

This section of *Integrative Systems Neuroscience* seeks to address some of the knowledge gaps through work that incorporates interdisciplinary and multiscale analyses of nervous systems. Integrative systems neuroscience represents the union of systems biology and integrative neuroscience.

Using a systems biology approach, the goal is to understand complexity by integrating disparate components to understand function as a whole, rather than merely the sum of individual parts. For example, in integrative neuroscience, this is accomplished by considering data from various biological levels to identify structure-function relationships and determine how subregions connect to enable higher-order emergent processes, such as behavior. Emergent properties are features of a complex system that are not evident in the individual components of a system in isolation and, therefore, cannot be predicted by only studying individual parts. Comprehensive models or computer simulations can be used to test how different biological components of the nervous system interact and contribute to these emergent processes. These models would ideally also account for the fact that many interactions amongst neural components are dynamic and may be non-linear in nature. And because science advances based upon models that make testable predictions, these models can be revised or refined based on new empirical data. Without models or theoretical frameworks in place, the potential hypothesis space becomes too large and hinders our ability to establish first principles that govern lower-level interactions.

A typical strategy for addressing potential knowledge gaps is to start with the data and go toward abstraction, such that you start from first principles and then ascend. But nervous systems are often so complex and dynamic that this cannot be accomplished logically. For example, if we have structural biology data that includes angstrom-level resolution to resolve the crystallography of individual proteins, such as ion channels, then how can we map this to a single neuron’s structure and function? How do we map microscopic information at the level of synapses and receptors to brain-wide circuits? As recent work using electron microscopy (EM) and machine learning algorithms has shown, a single neuron in the human cortex can have more than 5,000 individual synaptic connections, and there are hundreds of millions of synapses within a single cubic millimeter of cortical tissue (Shapson-Coe et al., 2024). Therefore, the scale of the structural datasets quickly becomes enormous at the microscopic level. Furthermore, this large amount of structural EM data does not include any dynamic functional information, such as synaptic activity that would vary over time at many, if not all, of the synapses. The scale of the complexity, if we were to include all these variables, impedes facile interpretation. Additionally, if we approach the problem from the “top-down” (e.g., circuits to proteins), we also quickly encounter large gaps in our understanding before we reach the level of individual neurons. These knowledge gaps are where a systems biology approach can leverage large amounts of quantitative information across different levels to draw important insights.

**FIGURE 1:**
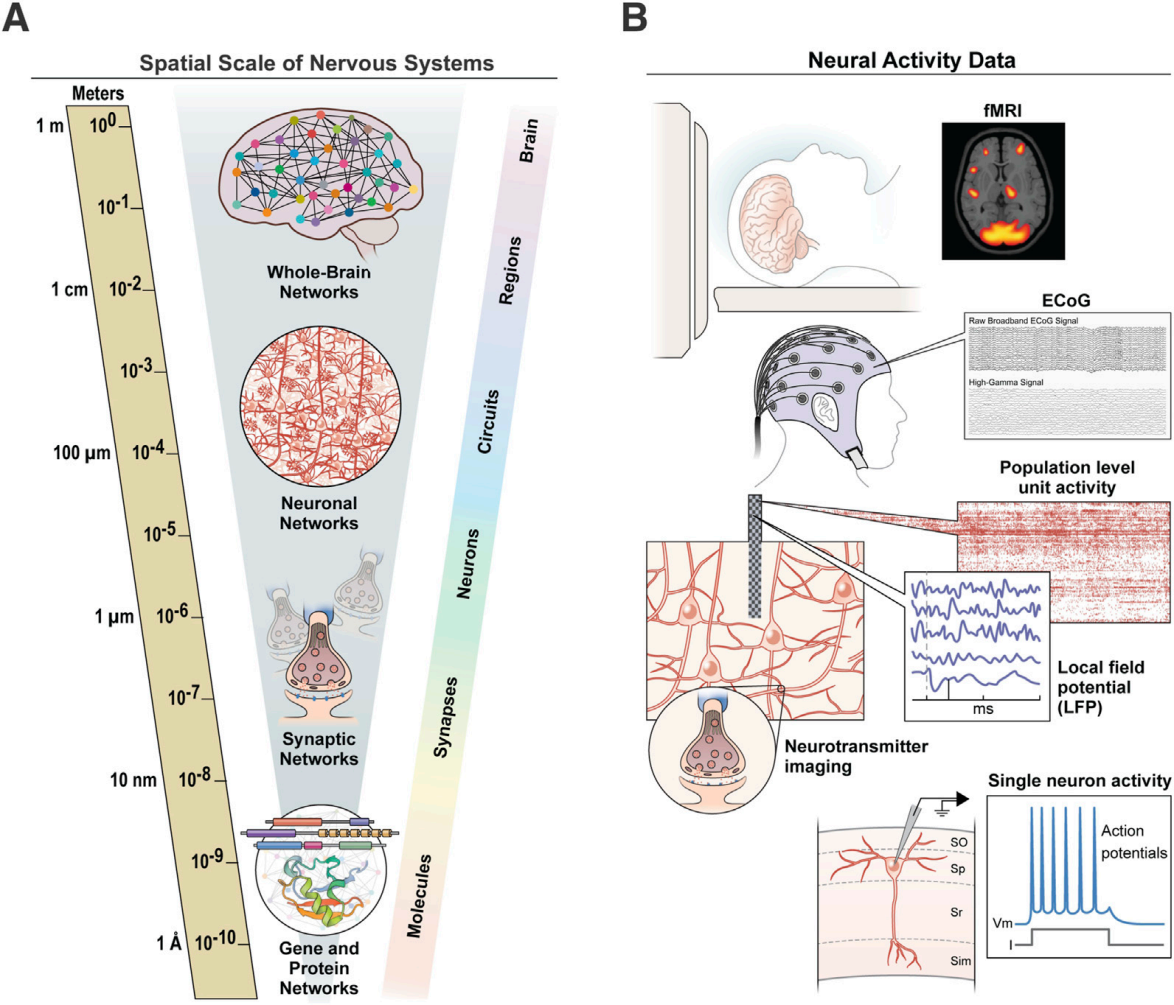
Spatial scale of nervous systems. **(A)** Nervous systems span many spatial scales, from molecules at the sub-micron level to whole-brain networks that span centimeters or more. **(B)** Each level of the nervous system requires distinct imaging and recording modalities to measure its relevant neural activity, presenting challenges to integration across modalities.

One particular challenge for integrating information in nervous systems is that the constitutive components operate over many orders of magnitude, at least six in the spatial domain (Figure 1A) and at least nine in the temporal domain (e.g., milliseconds to years; Lichtman and Denk, 2011). As a result, we are often left with gaps in knowledge between several spatiotemporal scales and domains that preclude a more general holistic understanding of regional function or whole organism behavior. Additionally, each scale often requires different modalities to image or record the relevant neural activity, presenting additional obstacles to functional integration (Figure 1B). One strategy to span some of these divides is to perform multimodal assessments in the same animals or subjects to examine the “ground truth” of what different signals mean at the biological and molecular levels (Caplan et al., 2011; Foxley et al., 2021). These types of ground truth studies give us information that can be directly measured or observed, rather than obtained through indirect measures. For example, in functional magnetic resonance imaging (MRI) studies, the blood-oxygen-level-dependent (BOLD) signal reflects changes in the oxygen level of the blood within a region of the brain. This BOLD signal is associated with changes in brain activity, but it is only a surrogate for the actual neural activity. Multimodal ground truth studies performed in non-human primates to specifically address this issue showed that the BOLD signal does, in fact, reflect changes in the local field potential within specific frequency bands (Goense and Logothetis, 2008; Logothetis et al., 2001). While the specifics of this correlation and the precise molecular mechanisms underlying the signals are still debated (Turner, 2016), these multimodal types of studies allow us to connect the different levels and scales of nervous system organization (Figure 1B). In some of my lab’s own research, we have used these within-sample approaches to combine widely used noninvasive imaging modalities (e.g. diffusion tensor imaging) with markers of what they are assumed to measure at the cellular and molecular level to assess how well these measures align with one another (Chang et al., 2016; Chang et al., 2017). Other studies have combined multiple imaging modalities spanning up to five spatial scales to perform within-sample comparisons of millimeter-resolution MRI data to nanometer-resolution EM data (Allegra Mascaro et al., 2015; Foxley et al., 2021).

The development of novel tools for precise neural circuit manipulation, such as optogenetics and chemogenetics, has elevated our ability to explore causal connections between dynamic activity patterns and specific behavioral outputs (Alexander et al., 2009; Rajasethupathy et al., 2016). Experiments combining these cell-type specific manipulations with multiple neural recording modalities, especially during animal behavior, have advanced our mechanistic understanding of neural systems-level processes (Adam et al., 2019; Fan et al., 2023). While these tools for manipulating genetically-defined circuits are powerful for going beyond simple correlational evidence, one should keep in mind that disrupting or intervening in a circuit does not necessarily explain how that circuit produced the specific behavior or function (Bickle, 2015; Wolff and Ölveczky, 2018). This is particularly true if that function is distributed across many large and diverse neural networks, each with its own systems and principles of operation. Of course, a primary feature of nervous systems is that many circuits are interconnected, so precise manipulations in one part of a circuit, even if transient, can produce unexpected effects in other parts of the system (Otchy et al., 2015). Such experimental outcomes further emphasize the need for developing integrative theoretical frameworks and mechanistic models that can help us make sense of emerging empirical datasets.

### Omic approaches and connectomes

The advent of omics technologies such as genomics, transcriptomics, proteomics, and metabolomics (known as the “Big Four”; Dai and Shen, 2022) has transformed the landscape of biological research. Starting in the 1990s, high-throughput DNA sequencing and mass spectroscopy introduced a new generation of large quantitative datasets that could systematically capture genetic and molecular changes with high accuracy. For example, by using genomic approaches, scientists can identify specific genes and genetic variations associated with disease conditions, enabling the development of targeted personalized therapies and interventions based on the biology of a specific individual (Hood et al., 2004). One aspect of these large-scale omics approaches is that they are mostly hypothesis-free, which can be advantageous in avoiding scientific biases. However, this unbiased approach could be a double-edged sword because it may be difficult to interpret or understand the functional significance of some of these very large datasets. While functional genomics and techniques, such as single-cell RNA sequencing and spatial transcriptomics, are starting to gain traction in neuroscience (Bhattacherjee et al., 2023; Jung and Kim, 2023), the adoption of these big data omic approaches has lagged behind that of other fields. This may be due in part to a reluctance by neuroscientists to engage in studies that do not have clearly defined hypotheses or because there is a lack of systems-level models to properly frame the resulting large datasets (Geschwind and Konopka, 2009).

**FIGURE 2:**
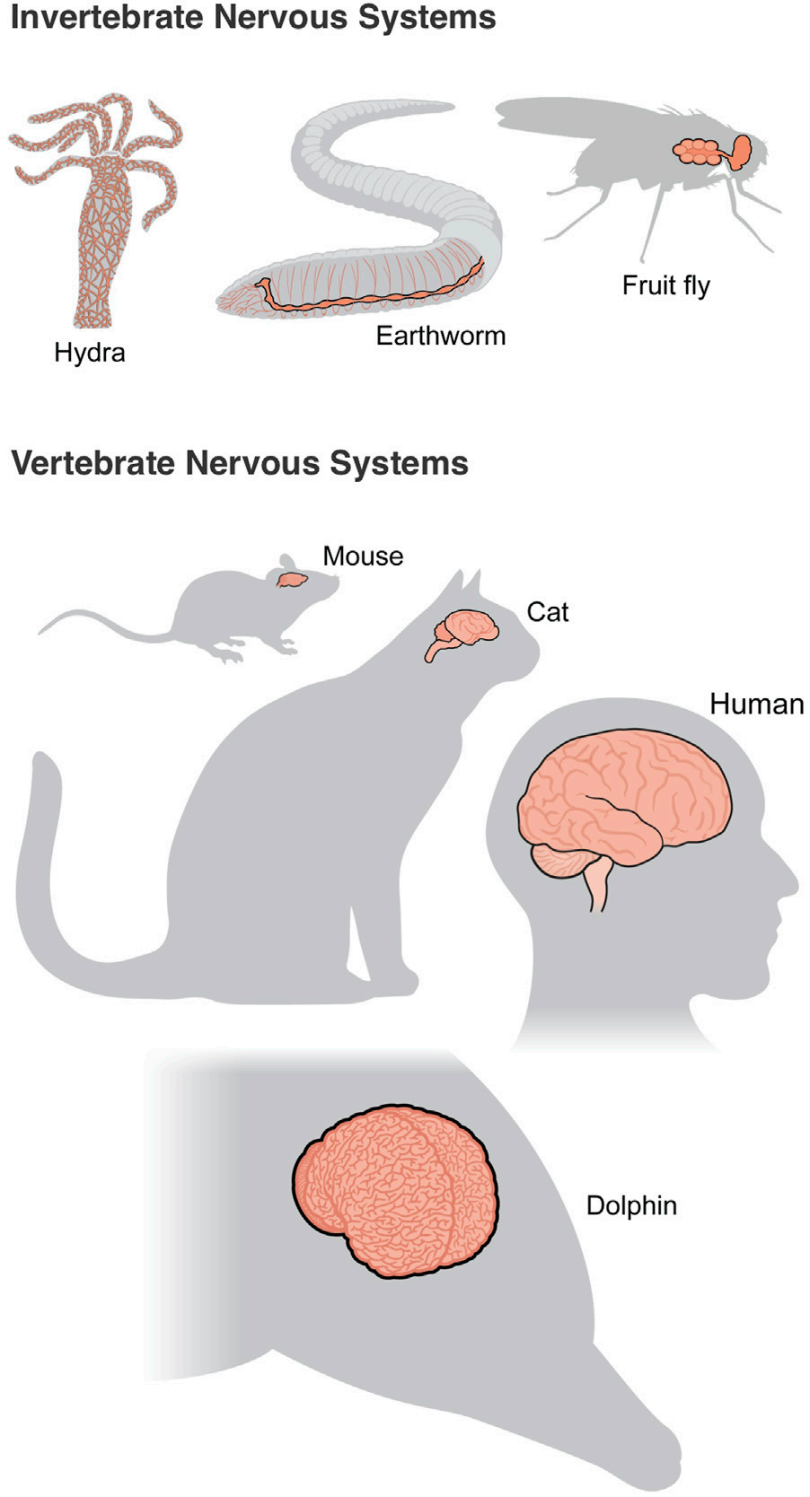
Different types of nervous systems. Among invertebrates, simple diffuse nerve nets in Hydra have connected neurons that span the organism’s body with no signs of centralization. Insects, such as earthworms and flies, have a brain and nerve cord with distributed ganglia. Amongst vertebrates, nervous systems (only brains are shown for simplicity) tend to be more centralized, complex, and specialized. As species evolved, more neurons were devoted to the neocortex to subserve higher-order processes. Notably, the human brain does not have the most number of neurons, as the brains of dolphins and whales actually have a higher amount.

One specific omics approach that is particularly relevant to neuroscience is connectomics. This approach aims to comprehensively map the synaptic connections between neurons within a piece of neural tissue or an entire organism’s nervous system (Lichtman et al., 2014). Connectomics displays the anatomic hard wiring that underlies information processing and, as such, provides important ground truth data for computational models and simulations. Because the synaptic connections between neurons can only be resolved at the nanometer scale, EM is currently the standard approach for collecting this information, but the acquisition, reconstruction, and error-free labeling of EM datasets is notoriously challenging. Up until recently, neuroscientists had only mapped the full connectomes of a handful of organisms: the nematode roundworm *Caenorhabditis Elegans*, the larva of the sea squirt *Ciona intestinalis*, and the marine annelid *Platynereis dumerilii*. The roundworm is the most complex of these, with 302 neurons making approximately 7,000 connections (White et al., 1986). However, very recently the connectome of the fruit fly *Drosophila melanogaster* has been completed in a larval brain (Winding et al., 2023) and an adult brain (Dorkenwald et al., 2024). These are major accomplishments for the field as fruit flies are capable of many sophisticated behaviors and their brains are orders of magnitude more complex than that of nematode roundworms. The adult fruit fly brain contains about 140,000 neurons and over 50 million synapses, all contained within a structure less than 1 mm wide. For comparison, a mouse brain has about 70 million neurons, and the human brain has 86 billion neurons, so mapping the connectome of higher animal species will require major advances in technologies and strategies (Figure 2). Nonetheless, the full connectivity diagram for these simpler species allows for structure-function models to be constructed of the full network architecture, which has been done in the roundworm brain (Brittin et al., 2021) and has enabled certain functional connectivity predictions in the fruit fly hemibrain (Turner et al., 2021; Scheffer et al., 2020). Already, computational models of the full fruit fly brain have been developed to understand sensorimotor processing with experimentally testable hypotheses (Shiu et al., 2024). Moreover, having these comprehensive wiring diagrams allows scientists to predict phenomena based on the connectivity data alone, which has now been demonstrated in a part of the fruit fly visual system (Seung, 2024). Predicting visual function from the underlying neural structure is important in fruit flies, as most of its brain is dedicated to vision. Additional work analyzing these connectome datasets will allow scientists to eventually perform full brain simulations based on mapped neuroanatomical data down to the level of individual neurons, axons, and dendrites. The broader aspiration of this approach is that connectomics will yield details about the underlying logic of neural wiring that can then be used to understand how neurons are connected with one another to drive systems-level processes.

Connectomics has allowed scientists to visualize and understand the nervous system at unprecedented levels of detail. However, many open questions and challenges remain as an organism’s connectome is not a static map. For example, synaptic connections are not all equal in weight, and there are important differences between structurally similar synaptic connections that are not visible through connectomics. In fact, the structural mechanism for memory formation in the brain is dependent on the plasticity of synaptic connections (Borczyk et al., 2021; Lamprecht and LeDoux, 2004). So if synapses are changing all the time, throughout an organism’s life, how can this structural plasticity be accounted for within connectomes? Moreover, while connectomics produces the complete wiring diagram of nervous systems, there is also wireless communication. A recent study in nematode roundworms showed that wireless signaling occurs through a neuropeptide network that is remarkably dense and has important effects on neural circuit function (Ripoll-Sánchez et al., 2023). In addition to neuropeptides, many other forms of communication occur within the nervous system that would not be captured in a synaptic connectome, including signaling by hormones, endorphins, gap junctions, or glial cells. To fully appreciate structure-function relationships, we need to have the dynamic real-time neural activity data to know what information is being communicated along the wires, as well as outside of the wires. These types of studies are already underway and will hopefully serve to provide important structure-function relationships for existing and future connectomes (Randi et al., 2023). There are substantial challenges to overcome in connectomics, however, having large volumes of digitized brain tissue at EM resolution will allow scientists to answer many questions about structure and connectivity in great detail (Morgan and Lichtman, 2017).

No matter what one generally thinks of connectomics and other big data omic approaches to solving biological problems, these high-throughput techniques can identify biologically relevant targets or patterns that can then be interrogated separately in hypothesis-driven experiments. By integrating data from multiple omics approaches, neuroscientists and systems biologists can create sophisticated foundational models that connect genetic and molecular information with the structural and functional properties of nervous systems.

### Emergence and convergence

While many people may instinctively assume that the main goal of neuroscience is to understand the human brain, there is, in fact, much to learn from much simpler nervous systems. The sheer diversity of nervous systems found within biology tells us that there are many different neural solutions to the various environments in which different organisms live. When considering the functions or goals of specific neural circuits at the systems level, we can look to lower animal species to glean insights into how evolution has designed specific solutions. For example, the original action potential work from Alan Hodgkin and Andrew Huxley was performed on the squid giant axon and revealed the general basic unit of functional communication, the action potential, used by neurons (Hodkin and Huxley, 1952). Incredibly, action potentials in the squid are more or less the same as those in a grasshopper, mouse, or human. Similarly, early foundational work on the visual system was conducted on photoreceptors from one of the oldest animals on earth, the horseshoe crab *Limulus polyphemus* (Fahrenbach, 1975). Many of the fundamental principles of learning and memory that we assume operate similarly in mammals were originally discovered and demonstrated in the sea slug *Aplysia* (Castellucci and Kandel, 1976; Kandel et al., 2014). The comparative approach is an important but often neglected sub-field in neuroscience that has the potential to unveil general principles in “lower” neural systems that remain relevant within higher-evolved animals.

Across the animal kingdom, there are many different body plans and accordingly, nervous system designs (Figure 2). While vertebrates typically have a central nervous system with a brain, invertebrates and other lower animal species differ widely in their nervous system organization. For example, the cephalopod octopus has a central brain structure in its body, but it actually has a total of nine different brains with one in each of its eight arms. And, the axial nerve cord at the center of each arm contains many more neurons than its central brain, which allows it to perform many complex coordinated motor movements and functions with nearly infinite degrees of freedom (Olson and Ragsdale, 2023). Recent work performing EM reconstruction of small portions of the octopus arm has identified new neural circuits and organizational features that enable some of these complex smooth movements that have long fascinated humans (Olson et al., 2024). Although these features are likely specialized for controlling octopus tentacles, the principles extracted from this work can have explanatory power in other nervous systems. Similarly, empirical studies in bats, barn owls, and zebra finches were all fundamental to our understanding of computational maps in the brain, which we use as a framework for understanding maps in other animal models (Laurent, 2020). As goal-directed spatial navigation is important for many animals, there are likely to be similarities in the spatial encoding of information amongst vertebrates, with potential comparable circuits in insects (Basu and Nagel, 2024). By taking a comparative approach to understanding how different nervous systems encode spatial representations, as one example, we can extract important common principles that have been conserved across evolution and also identify how differences between species may be linked to different species-specific behavioral or cognitive capacities.

In addition to maintaining a diversity of animal models to study, there should also be a focus on neural systems outside of the CNS and the brain so we can bring an improved understanding of physiology and function on the whole-organism level. Concerted efforts to study the peripheral and autonomic nervous systems in certain species are already underway in mammals (https://sparc.science), and lab groups are beginning to reveal important organizing principles about the structure, function, and genetics of peripheral neural circuits, such as the vagus nerve (Huerta et al., 2023; Prescott and Liberles, 2022; Zhao et al., 2022). Of course, the enteric nervous system (ENS), with its myriad cell types and functions, can be considered an integrative nervous system of its own. As the ENS has its own set of reflexes, pattern generators, and autonomic processes, there are likely important systems-level principles that can be used to understand other parts of the nervous system (Furness et al., 2014; Sharkey and Mawe, 2023).

In our search for the basic organizing rules that govern neural circuits, there is still much to learn from animals with simpler nervous systems. Studies in other animal species, such as the zebrafish *Danio rerio*, have examined whole-brain and whole-body neural activity to obtain a more holistic understanding of neural activity patterns. Because zebrafish bodies are transparent, scientists are able to perform live optical imaging of neural activity using fluorescent indicators and various dyes. This intact *in vivo* imaging approach is valuable as zebrafish are a vertebrate model with substantial homology to the mammalian brain and can engage in relatively sophisticated behaviors (Cong et al., 2017; Hasani et al., 2023). In fact, recent work has shown that zebrafish can compute a 3D model of their spatial environment, a visual perception ability that was previously thought to only exist in animals with more complex nervous systems (Zwaka et al., 2022). Since zebrafish perform sophisticated perceptual computations and their bodies can be imaged entirely intact, they represent a promising model system where multiple levels (e.g., behavior, circuits, neurons, whole-body physiology) can be simultaneously assessed for the purposes of integration.

### Technology and teams

“Progress in science depends on new techniques, new discoveries and new ideas, probably in that order”

– Sydney Brenner, PhD. 2002 Nobel Laureate in Physiology or Medicine

Just as technological innovations drove the prior decades of neuroscience discoveries, there is similarly a new era of advances tied to developments in modern computing which have been a driving force behind progress in systems biology and integrative systems neuroscience (Kanter et al., 2022; Yuste, 2015). High-throughput sequencing, advanced neuroimaging techniques, and powerful computational tools have enabled the collection and analysis of vast amounts of data, propelling our understanding of neural circuits to new heights. Moreover, artificial intelligence and machine learning algorithms have played a pivotal role in making sense of the enormous datasets generated by omics approaches, including for connectomics. A recent study mapped a single cubic millimeter of human cortical tissue at EM resolution, containing 57,000 cells and 150 million synapses that were individually segmented and labeled using machine-learning algorithms to produce high-resolution 3D rendering (Shapson-Coe et al., 2024). The resulting dataset was 1.4 *petabytes* (1,000 terabytes) in size, and this was only a single cubic millimeter of human brain tissue. Since the whole human brain is estimated to have more than a quadrillion (10^15^) synaptic connections, it is very likely that future connectomic datasets will be exabytes (1,000 petabytes) or larger in size. The immense scale of these datasets necessitates advanced computing approaches to even manage, let alone extract useful understanding from. But this is where machine learning and artificial intelligence can help humans to identify new patterns in complex multidimensional data that may not have been uncovered using traditional scientific approaches. For example, machine learning algorithms have been incorporated into various brain-computer interface devices to decode human brain activity, producing impressive outcomes in neural prostheses for human vision (Borda and Ghezzi, 2022) and language (Metzger et al., 2024; Silva et al., 2024). The incorporation of machine learning in biology and medicine has already led to significant advances in how we decode physiological signals and diagnose certain neurological diseases (MacEachern and Forkert, 2021; Myszczynska et al., 2020). As neuroscience datasets continue to grow in size and artificial intelligence-based tools continue to evolve rapidly, it is very likely that the next major discoveries in integrative systems neuroscience will be machine-assisted.

In 2020, a group of neuroscientists reflected upon the past 5 decades of neuroscience research and what they expect to see from the field in the next 5 decades (Altimus et al., 2020). One takeaway message is that modern neuroscience as a field is extremely vast and we must ask ourselves how to best study this vast “system of systems”. The best course is likely through interdisciplinary collaborations integrating the expertise of mathematicians, engineers, and biologists. Moreover, the next-generation of neuroscientists should be trained to be integrative and learn different methods that span scales, as well as fields (Buhusi et al., 2023; Grillner et al., 2005). Multidisciplinary multi-institutional collaborative efforts are already ongoing with projects such as the International Brain Laboratory, which “joins together diverse experimental and theoretical neuroscience teams to pursue a common goal: to develop a unified brain-wide theory of complex behavior, at the neuronal level” (International Brain Laboratory and International Brain Laboratory, 2017). The Brain Research through Advancing Innovative Neurotechnologies Initiative Cell Census Network (BICCN) is another large interdisciplinary team consisting of hundreds of scientists with the goal of cataloging brain cell types across humans, non-human primates, and mice (Chiou et al., 2023; Siletti et al., 2023). Together with the many “Big Science” endeavors being pursued at the Allen Institute and the Chan Zuckerberg Initiative, there has been a clear shift towards large team-based approaches to answer some of the most complex fundamental questions in neuroscience. The fruit fly connectome project called FlyWire consisted of hundreds of researchers, including citizen scientists, spread out over 127 institutions working together to segment, label, and proofread imaging data that was processed by artificial intelligence-assisted automated pipelines (Dorkenwald et al., 2024). Importantly, the FlyWire team has made the fruit fly connectome data freely available and open to explore with multiple online resources and databases, allowing anyone with an internet connection to access the information. Other large institutions, such as the Allen Institute, have also embraced an Open Science policy to increase transparency and accelerate the rate of discovery (http://brain-map.org; Koch and Jones, 2016). This scale of teamwork, openness, and cross-disciplinary collaboration, in combination with machine-assisted automation, is the type of large-scale effort that can lead to major foundational discoveries.

## Conclusion

The *Integrative Systems Neuroscience* section of *Frontiers in Systems Biology* aims to be a journal destination for work that integrates findings across scales and disciplines to better understand the nervous system. In the spirit of a multidisciplinary approach and diverse viewpoints, the journal will include studies from a wide range of animal models and human subjects, in addition to computational work exploring explanatory frameworks. In this Specialty Grand Challenge article, I have highlighted only a handful of studies that reflect how we can pursue a better understanding of neural circuits through integration. It is my hope that new experimental approaches and frameworks will bring about new theories that will bridge the disciplines of systems biology and integrative neuroscience.

Modern high-throughput technologies and big data approaches will continue to provide us with massive troves of molecular, genetic, and neurophysiological information. But information is not the same as knowledge. The challenge now is to integrate these different levels and types of data to develop foundational models that move beyond descriptors and advance our understanding. As the neuroscientist David Marr famously observed, “Trying to understand perception by studying only neurons is like trying to understand bird flight by studying only feathers: It just cannot be done” (Marr, 1982). Marr’s work emphasized that describing phenomena at individual levels will only provide us with descriptions rather than explanations. If we limit our understanding to those individual levels, then we will have a very difficult time understanding any systems, let alone whole nervous systems.
